# There Is More Than Meets the Label: Rethinking Saturated Fat and Cardiovascular Health

**DOI:** 10.31662/jmaj.2025-0120

**Published:** 2025-04-04

**Authors:** Atsushi Mizuno

**Affiliations:** 1 Department of Cardiovascular Medicine, St. Luke’s International Hospital, Tokyo, Japan

**Keywords:** Nutrition, Saturated fatty acids, Lipid, Cardiovascular disease, Personalized, Genetic nutrition

The Dietary Reference Intakes are established by the Minister of Health, Labour and Welfare under Article 16-2 of the Health Promotion Act to promote public health, prevent lifestyle-related diseases, and provide guidelines on energy and nutrient intake through diet. These guidelines, first introduced in 2005, have been revised every five years, incorporating the latest domestic and international scientific evidence and updates to various clinical practice guidelines. After extensive scientific review through expert committees and working groups, the Dietary Reference Intakes for Japanese (2025 Edition) has been published.

The key components of the guidelines are structured into three sections:

1. Energy and Nutrients

2. Targeted Population Characteristics

3. The Relationship Between Energy/Nutrient Intake and the Prevention or Management of Lifestyle-Related Diseases and Functional Health

The primary focus is on energy and nutrient intake, based on nutritional science. Among these, saturated fatty acids (SFAs) have been identified as a critical nutrient affecting public health, along with total fat, cholesterol, sugars, and sodium, as stipulated by the Ministry of Health, Labour and Welfare. The basis for this classification is supported by meta-analyses of interventional studies, which have indicated a significant reduction in cardiovascular disease (CVD) incidence when SFA is replaced with polyunsaturated fatty acids (PUFAs) ^(1)^.

The relationship between SFA and CVD risk has been a subject of research for decades. In 1992, Mensink et al. ^(2)^ reported that replacing SFA with monounsaturated fatty acids (MUFA) or PUFA (Figure 1) was associated with lower low-density lipoprotein cholesterol levels and consequently a smaller risk of CVD. This evidence provided the foundation for dietary strategies aimed at reducing the proportion of energy derived from SFA.

**Figure 1.**
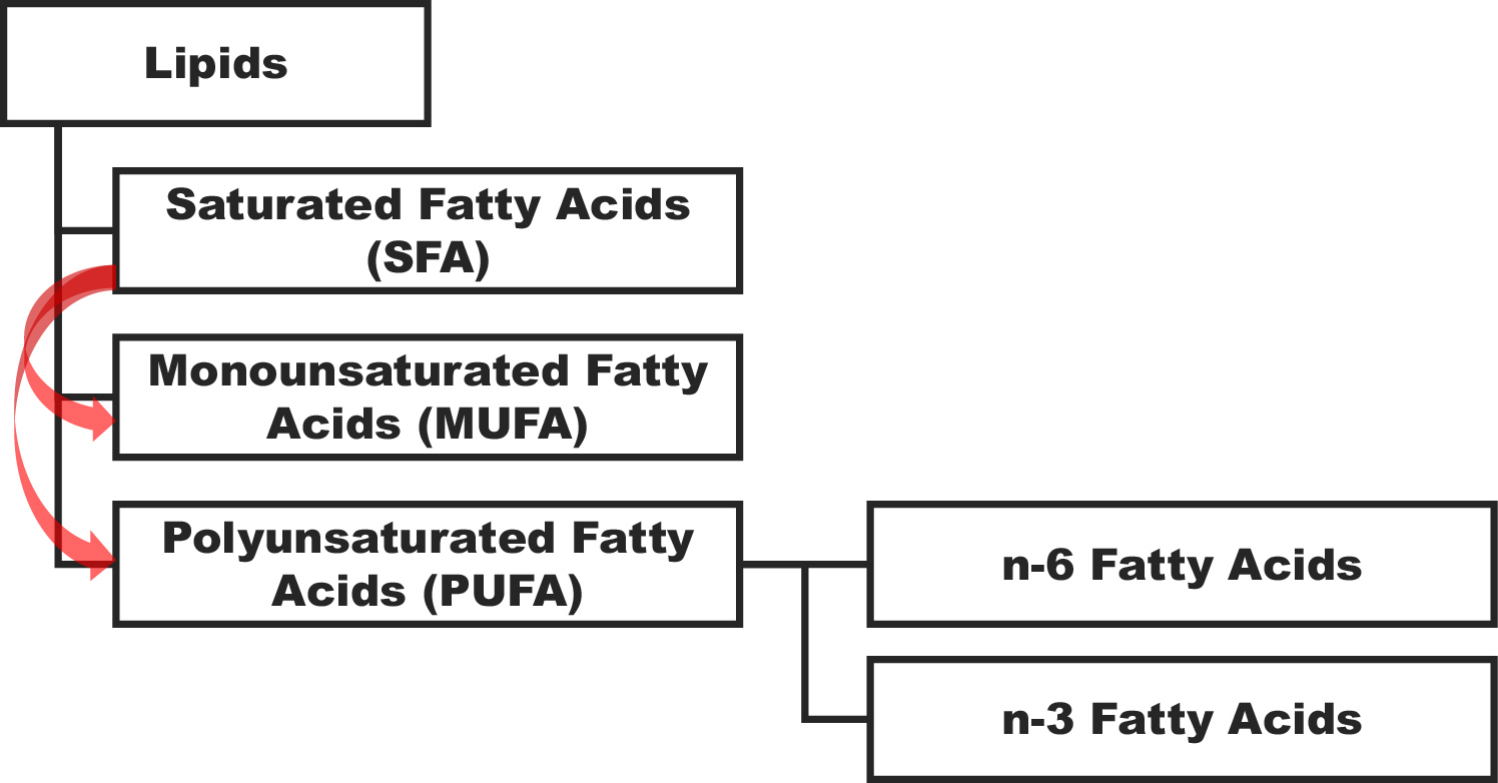
The classification of lipids into saturated Fatty acid (SFA) and unsaturated fatty acids. The red arrows represent the conventional dietary recommendation of replacing SFA with unsaturated fatty acids (MUFA and PUFA), a strategy historically linked to cardiovascular risk reduction based on metabolic and clinical evidence. MUFA: monounsaturated fatty acid; PUFA: polyunsaturated fatty acid.

However, in recent years, the linear association between SFA intake and CVD risk has been challenged. It has been argued that simply reducing SFA intake without considering the replacement nutrients or the specific food sources of SFA is unlikely to yield significant health benefits ^(3)^. A systematic review by Yamada et al. ^(4)^ analyzed nine randomized controlled trials (RCTs) involving a total of 13,532 participants and concluded that reducing SFA intake did not significantly reduce overall mortality, CVD-related mortality, myocardial infarction, or major cardiovascular events (excluding stroke) ^(4)^. Although the number of included studies was relatively small, and the research primarily focused on both primary and secondary prevention settings, this study contributes two important insights:

1. The efficacy of SFA reduction or replacement remains inconclusive owing to the lack of recent large-scale RCTs.

2. Future dietary strategies should take a more holistic approach by considering cultural and dietary differences, not only in terms of nutrient intake but also in the broader context of dietary patterns, food sources, and overall lifestyle when designing intervention trials, rather than applying a one-size-fits-all approach.

In the realm of secondary prevention, trials such as REDUCE-IT and RESPECT-EPA have shown that supplementing with n-3 PUFAs (n-3 PUFA) can reduce cardiovascular events ^(5)^. This raises the question of whether replacing SFA with PUFA is the optimal approach or whether supplementing with n-3 PUFA itself provides greater benefit. The debate has shifted from simple nutrient replacement strategies toward a more comprehensive approach that accounts for total energy intake, overall dietary patterns, and metabolic balance.

We are at a pivotal moment in redefining the future of nutrition science. Key areas of focus include ultra-processed foods (UPF), sustainability in animal-source foods, life-course nutrition, and the emerging field of personalized nutrition. UPFs have become a major concern owing to their evolving classification and growing evidence linking them to metabolic disorders and CVD. Rather than focusing solely on individual nutrients such as saturated fat, discussions now emphasize food processing methods and the classification of final food products. UPFs, characterized by refined ingredients and industrial additives, contribute to poor diet quality, chronic inflammation, and increased CVD risk. Their health impact extends beyond nutrient composition, highlighting the importance of processing and food structure. Sustainability in animal-source foods is increasingly debated, balancing nutritional adequacy with environmental concerns. Although it provides essential nutrients, livestock production increases climate and resource challenges. Strategies for sustainable diets include optimizing meat consumption, integrating plant-based proteins, and exploring alternative protein sources. Life-course nutrition shifts from nutrient-centric guidelines to holistic dietary approaches that consider age, food accessibility, and cultural factors. Recognizing ways nutrition affects long-term health, this perspective moves beyond standardized recommendations toward context-specific dietary guidance. Emerging research in personalized and precision nutrition aims to tailor dietary recommendations on the basis of genetic and metabolic differences. Although promising, its practical application, feasibility, and ethical concerns remain unresolved.

As nutrition policy evolves, it must balance evidence-based, population-wide guidelines with emerging insights into individualized nutrition. This study highlights the need for a more nuanced approach, integrating dietary patterns, sustainability, and personalization into future research and public health strategies.

## Article Information

### Conflicts of Interest

None

### Disclaimer

Atsushi Mizuno is one of the Editors of JMA Journal and on the journal’s Editorial Staff. He was not involved in the editorial evaluation or decision to accept this article for publication at all.
